# Metal–Organic-Framework-Derived Nitrogen-Doped Carbon-Matrix-Encapsulating Co_0.5_Ni_0.5_ Alloy as a Bifunctional Oxygen Electrocatalyst for Zinc–Air Batteries

**DOI:** 10.3390/ma17112629

**Published:** 2024-05-29

**Authors:** Jinglin Liu, Lina Han, Shicai Xiao, Anqi Zhu, Yingjie Zhang, Xiaoyuan Zeng, Peng Dong

**Affiliations:** 1 Faculty of Materials Science and Engineering, Kunming University of Science and Technology, Kunming 650031, China; 15614122661@163.com (J.L.); xsc13550226121@163.com (S.X.); zaq663146@163.com (A.Z.); zhangyingjie09@126.com (Y.Z.); zengxiaoyuan@kust.edu.cn (X.Z.); 2 National and Local Joint Engineering Research Center of Lithium-Ion Batteries and Materials Preparation Technology, Key Laboratory of Advanced Battery Materials of Yunnan Province, Kunming University of Science and Technology, Kunming 650031, China

**Keywords:** metal–organic frameworks, CoNi alloy, oxygen reduction reaction, Zn–air battery

## Abstract

The development of low-cost, high-performance oxygen electrocatalysts is of great significance for energy conversion and storage. As a potential substitute for precious metal electrocatalysts, the construction of efficient and cost-effective oxygen electrocatalysts is conducive to promoting the widespread application of zinc–air batteries. Herein, Co_x_Ni_y_MOF nanoparticles encapsulated within a carbon matrix were synthesized and employed as cathode catalysts in zinc–air batteries. Co_0.5_Ni_0.5_MOF exhibits superior oxygen reduction reaction (ORR) and oxygen evolution reaction (OER) performance and durability. The zinc–air battery assembled with Co_0.5_Ni_0.5_MOF as the air cathode exhibits a maximum power density of 138.6 mW·cm^−2^. These improvements are mainly attributed to the optimized metal composition of the cobalt–nickel alloy, which increases the specific surface area of the material and optimizes its pore structure. Significantly, the optimization of the electronic structure and active sites within the material has led to amplified ORR/OER activity and better zinc–air battery performance. This study underscores the immense promise of Co_0.5_Ni_0.5_MOF catalysts as feasible substitutes for commercial Pt/C catalysts in zinc–air batteries.

## 1. Introduction

Fossil fuels constitute non-renewable energy resources, and their reserves are gradually depleting owing to their extensive extraction and utilization by human beings [1]. Hence, the development of new energy sources has become paramount [2]. However, renewable energy sources such as solar and wind energy possess inherent intermittency, which underscores the significance of energy storage technologies [3]. Among numerous energy storage technologies, zinc–air batteries stand out due to their high theoretical energy density of 1320 Wh·kg^−1^ [4]. Zinc–air batteries, which utilize zinc as the anode material and an air electrode as the cathode [5], are completely free of toxic and harmful substances such as lead, cadmium, and mercury, and are thus considered environmentally friendly batteries [6]. Simultaneously, the manufacturing materials required for zinc-air batteries are easily available and the manufacturing process is simple, so the production cost is relatively low [7]. This gives them a notable competitive edge in large-scale battery applications [8].

Despite the numerous potential advantages of zinc–air batteries, their large-scale application remains constrained by several limitations [9]. The principal cause is rooted in the kinetically sluggish oxygen reduction reaction (ORR) and oxygen evolution reaction (OER) that take place at the air cathode. To address this issue, the use of high-performance bifunctional catalysts is necessary [10]. Currently, Pt/C catalysts have been commercialized primarily due to their exceptional performance in ORR, while RuO_2_ and IrO_2_ catalysts have similarly been commercialized for their performance in OER [11]. Nonetheless, the elevated cost of precious metal catalysts poses a significant impediment to the widespread adoption of zinc–air batteries. Consequently, the quest for cost-effective, high-performance bifunctional catalysts has emerged as a pivotal research endeavour [12]. Previously, alloying strategies have been widely used to improve the performance of noble-metal-based catalysts. The main principle is to modulate the adsorption capacity of intermediate containing substances by changing their spin states through coupling between dissimilar metals. In addition, in practical electrocatalytic applications, which involve strong acid or alkaline media solutions, it is difficult for pure metals to resist the corrosive nature of these media solutions, which makes it difficult for them to become efficient catalysts. Metal alloying is the easiest way to improve stability. Transition metal alloying can change the components of catalytically active metals, increase the degree of nanosizing, optimize d-band centres, and regulate the binding energy of metal catalytic sites and intermediates, thus effectively improving the electrocatalytic activity of transition metals.

Metal–organic framework materials (MOFs) are considered to have the potential to replace precious metal catalysts due to their excellent structural tunability and diversity, ease of compositing and modification, and environmental friendliness [13]. Duan et al. grew ultrathin 2D Ni-Fe-based MOF nanosheet arrays in situ on different substrates using a chemical liquid deposition method [14]. The synthesized material possesses an ultrathin nanosheet configuration, excellent conductivity, and a hierarchical pore structure, resulting in significantly enhanced catalytic performance with high activity towards OER, HER, and overall water splitting. Aijaz et al. reported another unique ZIF-67-derived composite material. The composite material consists of a Co@Co_3_O_4_ core–shell structure formed by N-doped carbon polyhedrons synthesized through reductive carbonization in a H_2_ atmosphere to produce this novel hybrid [15]. Compared to IrO_2_ and RuO_2_, this inexpensive catalyst exhibits superior electrocatalytic activity. The Loh research group prepared a GO-encapsulated Cu-based MOF composite for use as a trifunctional catalyst for OER, HER, and ORR. Due to its unique porous structure, good charge transport properties, and the synergistic effect between graphene oxide and MOFs, the GO/Cu-MOF material exhibits a lower overpotential and higher current density compared to pure MOFs when catalysing OER, HER, and ORR under acidic conditions [16]. However, current MOF catalysts still suffer from issues such as poor stability and weak conductivity [17]. To address these issues, in this work, nitrogen-doped carbon-matrix-encapsulating CoNi alloy nanoparticles were synthesized. These catalysts exhibit high oxygen reduction and oxygen evolution performance. Experimental findings reveal that the synergistic effect between the bimetals contributes to the optimized electronic structure among the metals. Additionally, varying the metal ratio successfully refined the specific surface area and pore volume. The synthesized Co_0.5_Ni_0.5_MOF catalyst exhibited outstanding ORR and OER activity in alkaline electrolytes, with an onset potential of 0.90 V, a half-wave potential of 0.82 V, and a limiting current density of 4.0 mA·cm^−2^. The zinc–air battery with Co_0.5_Ni_0.5_MOF as the air cathode exhibits the highest power density and energy density. More importantly, there is almost no change in the charge–discharge voltage difference after 200 h of charge–discharge cycling at a current density of 5 mA·cm^−2^.

## 2. Experiment

### Preparation of Co_x_Ni_y_MOF

Take the preparation process of the Co_0.5_Ni_0.5_MOF catalyst as an example. Cobalt nitrate (0.815 g Co(NO_3_)_2_·6H_2_O. Aladdin, Shanghai, China) and nickel nitrate (0.814 g Ni(NO_3_)_2_·6H_2_O. Aladdin, Shanghai, China) were added to an appropriate amount of methanol (98 wt%. Aladdin, Shanghai, China) and mixed uniformly. Subsequently, the mixture was stirred at room temperature for 2 h at 350 rpm in a methanol solution containing 48 mM 2-methylimidazole (98 wt%. Aladdin, Shanghai, China). Afterwards, the mixture was allowed to stand for 24 h, followed by centrifugation (8000 rpm) and washing with methanol. The precursor was then obtained by vacuum-drying at 60 °C overnight. The 100 mg precursor was loaded into a crucible and annealed at 800 °C for 2 h under a nitrogen atmosphere (heating rate: 5 °C/min; nitrogen flow rate: 10 L/min), followed by natural cooling to room temperature. We named the resulting catalyst as Co_0.5_Ni_0.5_MOF. A series of samples were prepared in order to study the effect of different ratios of cobalt to nickel. The collected sample was denoted as Co_x_Ni_y_MOF (where x:y represents the ratio of cobalt to nickel metals). For comparison, Co_x_Ni_y_MOF catalysts with different cobalt-to-nickel ratios were prepared using the same method. The amount of drugs added in the experiment is shown in Table 1.

## 3. Results and Discussion

### 3.1. Preparation and Characterization of Co_x_Ni_y_MOF

**Figure 1:**
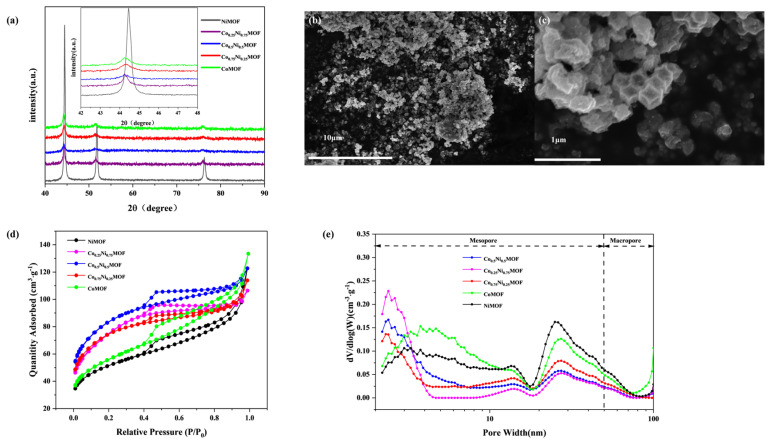
(**a**) XRD patterns of Co_x_Ni_y_MOF. (**b**,**c**) FESEM images of Co_0.5_Ni_0.5_MOF. (**d**) N_2_ adsorption_desorption isotherms and (**e**) the pore_size distribution of Co_x_Ni_y_MOF.

To investigate the structural properties of the synthesized catalysts, X-ray diffraction (XRD) patterns were investigated, as presented in Figure 1a. The diffraction peaks at 44.21°, 51.52°, and 75.85° correspond to the (111), (200), and (220) planes of the CoNi alloy, respectively, according to the standard PDF cards (PDF#15-0806, PDF#15-0850). Compared with CoMOF, the diffraction peaks gradually shift to the right as the proportion of metallic nickel gradually increases. This is because the radius of nickel ions is smaller than that of cobalt ions, so nickel doping makes the overall crystal plane spacing decrease, which causes the diffraction peaks to shift to the right.

The N_2_ adsorption–desorption isotherms for all catalysts are depicted in Figure 1. Upon analysis of the nitrogen adsorption and desorption curves presented in Figure 1c, a distinct hysteresis loop is evident, indicating the presence of numerous mesopores within the material. Co_0.5_Ni_0.5_MOF has the most excellent specific surface area of 278.5 m^2^/g, which is superior to CoMOF (196.5 m^2^/g), Co_0.75_Ni_0.25_MOF (246.0 m^2^/g), Co_0.25_Ni_0.75_MOF (262.2 m^2^/g), and NiMOF (171.1 m^2^/g). Co_0.5_Ni_0.5_MOF’s high specific surface area facilitates the exposure of catalytically active sites (Figure 1e). The presence of mesopores is important to enhance the catalytic properties of the materials. The pore-size distribution in Co_0.5_Ni_0.5_MOF catalysts is concentrated mainly in the range of 2–5 nm and 20–50 nm. The presence of mesopores with different pore sizes provides channels for oxygen transport, while the high specific surface area ensures sufficient active sites, which together contribute to the improvement of ORR performance [17].

FESEM and SEM techniques were utilized to investigate the structure and morphology of the synthesized catalysts, as shown in Figure 1b. The morphology of the Co_0.5_Ni_0.5_MOF catalyst is in the shape of a hexahedral framework, and this morphology is formed by the precursor after pyrolysis. This framework structure may contribute to the exposure of active sites. In addition to this, different degrees of agglomeration of the catalyst particles can be observed.

**Figure 2:**
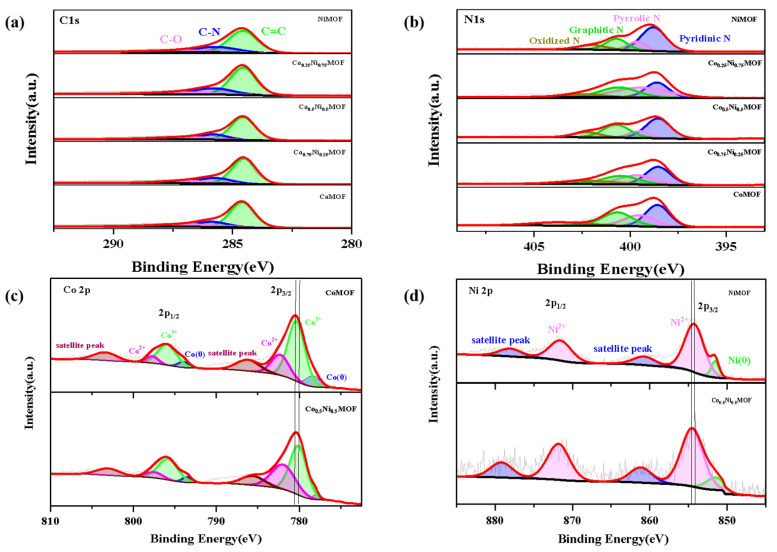
XPS spectra of (**a**) high-resolution C 1s of Co_x_Ni_y_MOF. (**b**) High-resolution N 1s of Co_x_Ni_y_MOF. (**c**) High_resolution Co 2p of Co_0.5_Ni_0.5_MOF and CoMOF. (**d**) High-resolution Ni 2p of Co_0.5_Ni_0.5_MOF and NiMOF.

The XPS survey spectrum of the Co_x_Ni_y_MOF catalyst reveals the presence of Co, C, N, O, and Ni elements, with the compositional analysis results displayed in Figure S1. The high-resolution spectra of C 1s indicate the presence of C–N bonds, suggesting that elemental N was successfully doped into the carbon matrix (in Figure 2a). The high-resolution spectra of N 1s show that there are four main types of N in the material: pyridine N (398.5 eV), pyrrole N (399.5 eV), graphite N (400.4 eV), and oxidized N (402.4 eV) (in Figure 2b). According to previous research results, it is widely recognized that pyridine N possesses the capability to accept electrons and serves as one of the active species in ORR, whereas graphite N is advantageous in improving the conductivity and structural stability of the material. Among the prepared catalysts, Co_0.5_Ni_0.5_MOF exhibited the highest combined content of pyridine N (35%) and pyrrole N (30%), demonstrating its superiority in terms of catalytic active site density.

The Co 2p peaks were fitted to the Co 2p_3/2_ peak (781.2 eV) and 2p_1/2_ peak (796.5 eV), and the Co 2p_3/2_ peak (778.7 eV) and 2p_1/2_ peak (793.5 eV), respectively. The Ni 2p peaks were fitted to the Ni 2p_3/2_ peak (857.2 eV) and the 2p_1/2_ peak (875.1 eV), respectively, and the Ni 2p_3/2_ peak (851.5 eV). Compared to CoMOF, the Co 2p of Co_0.5_Ni_0.5_MOF exhibits a negative shift. Similarly, in comparison to NiMOF, the Ni 2p of Co_0.5_Ni_0.5_MOF demonstrates a positive shift. It is known that the electronegativity of Co is stronger than that of Ni, indicating that nickel acts as an electron donor in the catalyst to transfer electrons to the cobalt site as an electron acceptor, optimizing the d-band centre of the material and regulating the adsorption capacity of the active sites of Co and Ni [18]. This enhances the suitability of the overall metal active sites for the adsorption and desorption of intermediates, thereby improving the catalytic performance of both ORR and OER [19].

### 3.2. ORR/OER Activity and Durability

**Figure 3:**
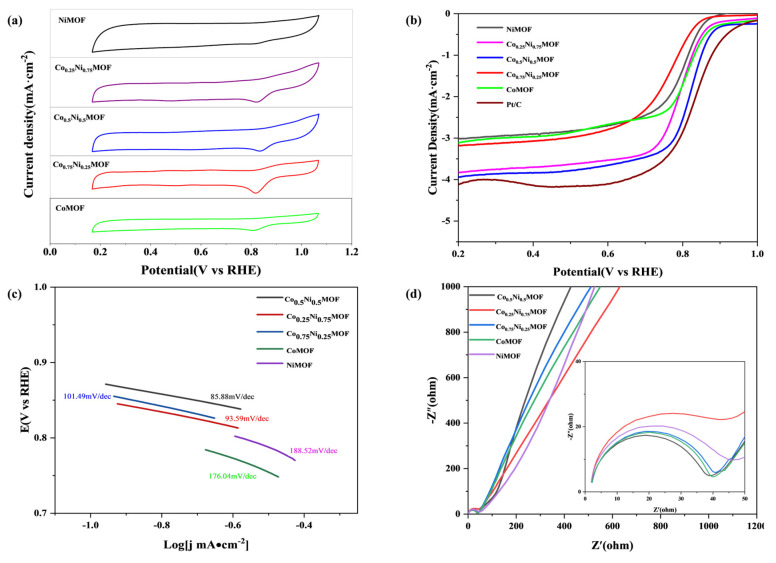
(**a**) CV curves for Co_x_Ni_y_MOF in O_2__saturated 0.1 M KOH solution with a scan rate of 50 mV s^−1^. (**b**) ORR LSV plots of the as-prepared catalysts. (**c**) ORR Tafel slopes obtained from the LSV plots of Co_x_Ni_y_MOF. (**d**) Nyquist plots of the aforementioned catalysts in an O_2__saturated 0.1 M KOH solution.

The electrochemical performance of the synthesized catalysts was assessed through the utilization of cyclic voltammetry (CV) and linear sweep voltammetry (LSV) curves, which were obtained via a rotating disk electrode (RDE) test. Firstly, as shown in Figure 3a, the CV test was performed under a 0.1 M KOH oxygen saturation condition, and the CV curves of all Co_x_Ni_y_MOF catalysts showed obvious redox peaks. The oxygen reduction peaks showed different positive shifts with iron doping, indicating that the performance of the iron-doped catalysts was improved to different degrees. Among them, the oxygen reduction peak position of Co_0.5_Ni_0.5_MOF had the highest potential of 0.83 V. This means that the oxygen reduction peak position of Co_0.5_Ni_0.5_MOF is closer to the standard potential of 1.23 V. The proximity of the peak positions is positively correlated with the ORR activity of the catalysts; therefore, the oxygen reduction peak positions of Co_0.5_Ni_0.5_MOF are closer to the standard potential, indicating a higher ORR activity [20]. The analysis of Figure 3b shows that adjusting the ratio of metal cobalt–nickel can effectively improve the electrocatalytic activity of the catalysts, and from the LSV results, when the ratio of metal cobalt–nickel is 1:1, the starting voltage, half-wave voltage, and limiting current of Co_0.5_Ni_0.5_MOF are 0.90 V, 0.82 V, and 4.0 mA·cm^−2^. The ORR performance of Co_0.5_Ni_0.5_MOF was the best among the Co_x_Ni_y_MOF catalysts [21].

The Tafel slope plays a crucial role in assessing the kinetic performance of the oxygen reduction reaction (ORR) (Figure 3c). Co_0.5_Ni_0.5_MOF has the lowest Tafel slope of 85.88 mV/dec, which is slightly lower than CoMOF (176.04 mV/dec), Co_0.75_Ni_0.25_MOF (101.49 mV/dec), Co_0.25_Ni_0.75_MOF (93.59 mV/dec), and NiMOF (188.52 mV/dec). This suggests that the kinetic reaction rate of Co_x_Ni_y_MOF catalysts can be enhanced by optimising the ratio between cobalt and nickel metals, which can be attributed to the excellent bimetallic synergistic effect between cobalt and nickel metals and the excellent electrical conductivity of MOF materials [22]. The electrochemical impedance (EIS) test results of the prepared Co_x_Ni_y_MOF catalysts are shown in Figure 3d. It can be seen that the radius of the semicircle of the Co_0.5_Ni_0.5_MOF catalyst is the smallest in the high-frequency region, which suggests that the Co_0.5_Ni_0.5_MOF catalyst has the smallest charge transfer resistance. In the low-frequency region, the Co_0.5_Ni_0.5_MOF catalyst has the largest slope, indicating that the catalyst has the smallest diffusion resistance. In conclusion, the Co_0.5_Ni_0.5_MOF catalyst has the highest electrical conductivity [23].

The overpotential at a current density of 10 mA·cm^−2^ is an important index for evaluating the OER activity of the catalysts (Figure S4). By comparison, it was found that the overpotential of Co_0.5_Ni_0.5_MOF (470 mV) was lower than that of CoMOF (600 mV), Co_0.25_Ni_0.75_MOF (520 mV), Co_0.75_Ni_0.25_MOF (500 mV), and NiMOF (540 mV). Co_0.5_Ni_0.5_MOF possessed better OER catalytic activity. The difference between the overpotential and the half-wave potential of the oxygen reduction reaction (ORR) is denoted as ∆E, which serves as a crucial metric for evaluating bifunctional catalytic activity. Co_0.5_Ni_0.5_MOF has the lowest ∆E (0.88 V), suggesting that it possesses a bifunctional electrocatalytic activity of ORR/OER that can be compared with that of noble-metal-based catalytic materials. Good bifunctional catalytic activity is important for the application of Co_0.5_Ni_0.5_MOF in rechargeable zinc–air batteries [24].

**Figure 4:**
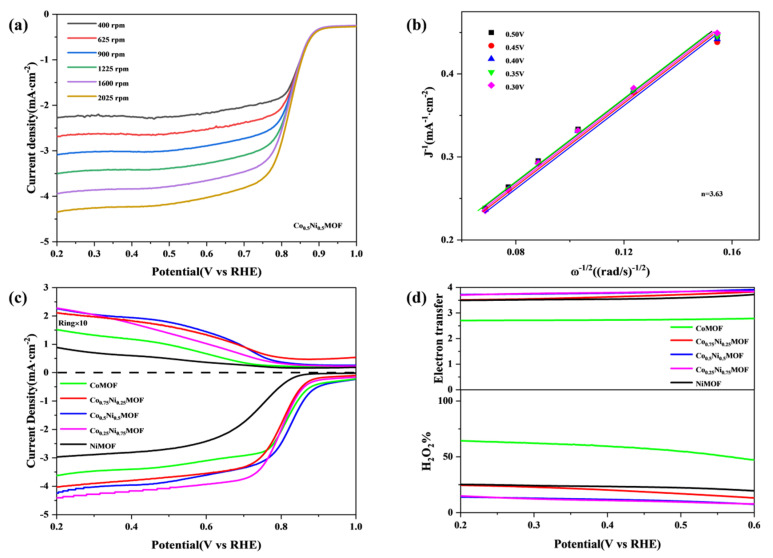
(**a**,**b**) LSV curves of Co_x_Ni_y_MOF at different rotation speeds and the K_L plots at different potentials. (**c**) RRDE data of Co_x_Ni_y_MOF. (**d**) The yield of hydrogen peroxide and the number of electron transfers.

To gain further insight into the reaction mechanism, the kinetics of the oxygen reduction reaction (ORR) were investigated in more detail using the Koutecky–Levich (K-L) equation, as illustrated in Figure 4a. The K-L plot shows a good linear relationship, which indicates that the kinetics of the primary reaction carried out on Co_x_Ni_y_MOF catalysts is related to the amount of dissolved oxygen in the electrolyte. We calculated the number of transferred electrons (n) based on the K-L equation, and the average number of transferred electrons for the Co_0.5_Ni_0.5_MOF catalyst was 3.63. The main ORR reaction pathway for Co_0.5_Ni_0.5_MOF catalysts is the four-electron reaction pathway. The other samples were also tested—CoMOF (2.40), Co_0.25_Ni_0.75_MOF (3.58), Co_0.75_Ni_0.25_MOF (3.52), and NiMOF (3.57)—and it was found that their ORR reaction pathways were a combination of two-electron and four-electron pathways [25].

RRDE experiments were conducted to further explore the electron transfer pathways and hydrogen peroxide (H_2_O_2_) yields of the Co_x_Ni_y_MOF catalysts. The results are depicted in Figure 4c,d. Because the intermediate product H_2_O_2_ is strongly oxidising, too much H_2_O_2_ will lead the catalytic activity of the catalyst to decay. The small H_2_O_2_ yield (≈12%) of the Co_0.5_Ni_0.5_MOF catalyst in the ORR-catalysed reaction indicates the high catalytic selectivity of the Co_0.5_Ni_0.5_MOF catalyst. The average number of transferred electrons calculated by the RRDE test is likewise the highest average number of transferred electrons for the Co_0.5_Ni_0.5_MOF catalyst, which is consistent with the conclusions drawn from the RDE test. This further suggests that the ORR reaction pathway of Co_0.5_Ni_0.5_MOF catalyst is mainly a four-electron reaction pathway [26].

### 3.3. Zn–Air Battery Performance

**Figure 5:**
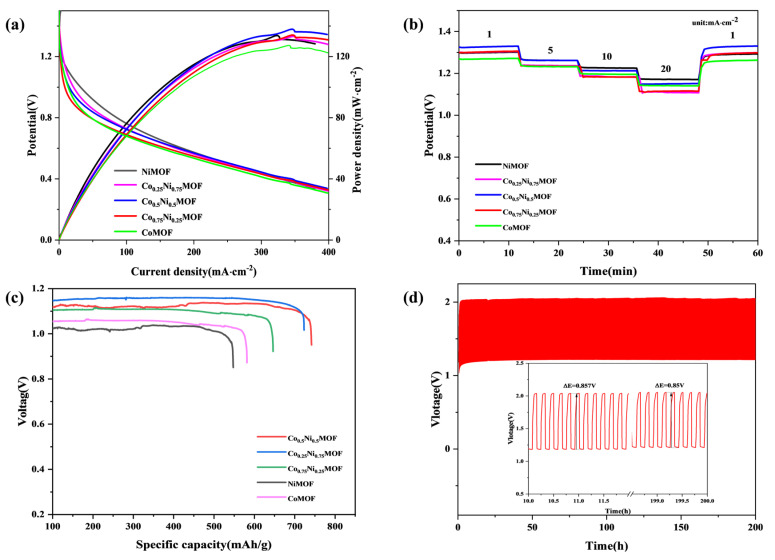
(**a**) Polarization and corresponding power density curves; (**b**) discharge curves of the Co_x_Ni_y_MOF-based Zn_air batteries; (**c**) galvanostatic discharge curves of the battery at different current densities; (**d**) charge_discharge curves at 5 mA cm^−2^ for the Co_0.5_Ni_0.5_MOF-based Zn–air batteries.

In order to further evaluate the practical application of Co_x_Ni_y_MOF catalysts, zinc–air batteries were assembled using Co_x_Ni_y_MOF catalysts as air cathode catalysts. In Figure 5a, the zinc–air battery with Co_0.5_Ni_0.5_MOF has the highest peak current density of 138.6 mW·cm^−2^, followed by CoMOF (127.9 mW·cm^−2^), Co_0.25_Ni_0.75_MOF (134.5 mW·cm^−2^), Co_0.75_Ni_0.25_MOF (134.2 mW·cm^−2^), and NiMOF (133.8 mW·cm^−2^). Furthermore, the rate capability of the Zn-air battery was evaluated through discharge curves. The results indicate that the Co_0.5_Ni_0.5_MOF-based zinc–air battery exhibits the highest stable voltage at lower current densities. Upon returning the current density to its initial value of 1 mA·cm^−2^, the discharge voltage reverted to its original level. The Co_0.5_Ni_0.5_MOF-based zinc–air battery has excellent stability and reversibility (Figure 5b).

After conducting a prolonged discharge test on the battery, it was observed that the Co_0.5_Ni_0.5_MOF electrode consistently maintained a stable discharge platform voltage of 1.13 V (in Figure 5c). The zinc–air battery catalysed by Co_0.5_Ni_0.5_MOF exhibits a normalized specific discharge capacity of 740 mA h g_Zn_^−1^, based on the mass of zinc. These values surpass those achieved by CoMOF (581 mA h g_Zn_^−1^), Co_0.75_Ni_0.25_MOF (648 mA h g_Zn_^−1^), Co_0.25_Ni_0.75_MOF (723 mA h g_Zn_^−1^), and NiMOF (549 mA h g_Zn_^−1^), highlighting the superior performance of the Co_0.5_Ni_0.5_MOF catalyst. Further evidence supporting the promising application prospects of the Co_0.5_Ni_0.5_MOF-based zinc–air battery is provided through battery stability testing (Figure 5d). After continuous operation for 200 h, the charge–discharge potential difference of the Co_0.5_Ni_0.5_MOF-based zinc–air battery remains around 0.85 V, demonstrating its exceptional cycling stability and outstanding ORR/OER performance. In conclusion, Co_0.5_Ni_0.5_MOF emerges as a high-performance bifunctional catalyst with potential for significant practical applications in rechargeable zinc–air batteries.

## 4. Conclusions

In summary, we have synthesized carbon-based encapsulated CoNi alloy nanoparticles and modulated the microstructure of Co_x_Ni_y_MOF by adjusting the ratio between the cobalt and nickel metals to enhance oxygen reduction reaction/oxygen evolution reaction performance. Through performance testing, the optimal Co_0.5_Ni_0.5_MOF catalyst exhibits exceptional onset potential, half-wave potential, limiting current density, and durability. By modifying the metal composition, the specific surface area of the material is augmented, and the pore structure of the material is refined. Additionally, the electronic structure of the material has undergone adjustment, promoting electron transfer and elevating the catalytic activity of the material. Meanwhile, the zinc–air battery assembled with the Co_0.5_Ni_0.5_MOF catalyst exhibits excellent power density and robust stability. Our synthetic strategy can provide insights for developing low-cost, high-stability catalysts for zinc–air batteries.

## Figures and Tables

**Table 1:** The molar ratios of cobalt nitrate and nickel nitrate used in the synthesis process of different samples.

	Cobalt Nitrate (g)	Nickel Nitrate (g)	2-Methylimidazole (g)
CoMOF	1.63	0	3.99
Co_0.75_Ni_0.25_MOF	1.222	0.407	3.99
Co_0.5_Ni_0.5_MOF	0.815	0.814	3.99
Co_0.25_Ni_0.75_MOF	0.407	1.221	3.99
NiMOF	0	1.628	3.99

## Data Availability

Data are contained within the article and Supplementary Materials.

## Supplementary Materials

The following supporting information can be downloaded at: https://www.mdpi.com/article/10.3390/ma17112629/s1, Figure S1: XPS spectra for CoxNiyMOF; Figure S2: High-resolution XPS O 1s spectra of CoxNiyMOF; Figure S3: Current–time (i-t) curves of CoxNiyMOF; Figure S4: Polarization curves of CoxNiyMOF and Pt/C recorded at a scan rate of 10 mV s^−1^ in 0.1 M KOH solution; Figure S5: Koutecky–Levich plots of the Co_0.25_Ni_0.75_MOF catalysts between 0.30 and 0.50 V; Figure S6: Koutecky–Levich plots of the Co_0.75_Ni_0.25_MOF catalysts between 0.30 and 0.50 V; Figure S7: Koutecky–Levich plots of the CoMOF catalysts between 0.30 and 0.50 V; Figure S8: Koutecky–Levich plots of the NiMOF catalysts between 0.30 and 0.50 V; Figure S9: Charge–discharge curves of the CoMOF Zn–air batteries at 5 mA cm^−2^; Figure S10: Charge–discharge curves of the Co_0.75_Ni_0.25_MOF Zn–air batteries at 5 mA cm^−2^; Figure S11: Charge–discharge curves of the Co_0.25_Ni_0.75_MOF Zn–air batteries at 5 mA cm^−2^; Figure S12: Schematic structure of Co_0.5_Ni_0.5_MOF catalysts; Figure S13: Schematic diagram of material structure and mechanism; Figure S14: SEM images of (a) CoMOF, (b) Co_0.75_Ni_0.25_MOF, (c) Co_0.25_Ni_0.75_MOF, and (d) NiMOF; Table S1: Summary of ORR and OER performance of CoxNiyMOF electrocatalysts; Table S2: The specific surface area of the prepared samples; Table S3: The prepared samples analyzed according to the battery performance of zinc–air batteries; Table S4: Parameters for the degassing process of nitrogen adsorption and desorption tests; Table S5: Setup parameters for the analytical process of nitrogen adsorption and desorption testing; Table S6: C, N, O, Co, and Ni content of each catalyst surface measured by XPS; Table S7: Relative content of C=C, C–N, and C–O bonds to elemental C in each catalyst surface; Table S8: C–OH and C=O bonds accounting for the relative content of elemental O in each catalyst surface; Table S9: The content of different types of N on the surface of the prepared catalysts obtained by XPS tests; Table S10: The molar ratios of cobalt nitrate and nickel nitrate used in the synthesis process of different samples; Table S11: The molar ratios of cobalt nitrate and nickel nitrate used in the synthesis process of different samples; Table S12: The specific surface area of pore volumes.
