# Supplementary material for: Metal–Organic-Framework-Derived Nitrogen-Doped Carbon-Matrix-Encapsulating Co0.5Ni0.5 Alloy as a Bifunctional Oxygen Electrocatalyst for Zinc–Air Batteries
Source: Materials (Basel). 2024 May 29;17(11):2629. doi: 10.3390/ma17112629 (PMC11173693; doi:10.3390/ma17112629)
Supplement: Supplementary file 1 [file materials-17-02629-s001.zip › materials-2968329-supplementary.pdf]

Supporting Information

Metal–organic-framework-derived nitrogen-doped carbon-matrix-encapsulating $Co_{0.5}Ni_{0.5}$ alloy as a bifunctional oxygen electrocatalyst for zinc－air batteries

Jinglin Liu[a,b], Lina Han[a,b,*], Shicai Xiao[a,b], Anqi Zhu[a,b], Yingjie Zhang[a,b], Xiaoyuan Zeng[a,b], Peng Dong[a,b,*]

[a] Faculty of Materials Science and Engineering, Kunming University of Science and Technology, Kunming 650000, PR China

[b] National and Local Joint Engineering Laboratory for Lithium-ion Batteries and Materials Preparation Technology, and Key Laboratory of Advanced Battery Materials of Yunnan Province, Kunming University of Science and Technology, Kunming 650000, PR China

* Corresponding author.

E-mail: hanln2016@163.com (L. Han);

1.Experimental Component

1.1 Material Preparation: Nickel nitrate hexahydrate ($Ni(NO_3)_2·6H_2O$, AR) was purchased from Aladdin. Cobalt nitrate hexahydrate ($Co(NO_3)_2·6H_2O$, AR) and 2-Methylimidazole (98%) were provided by Adamas-beta. Anhydrous methanol (Methyl Alcohol, GR, Anhydrous, ≥ 99.9%) was provided by General-reagent.Nafion (5.0 wt%) was purchased from Sigma-Aldrich. Ethanol (C_2H_5OH, AR) was bought from Fengchuan Chemical.

1.2 Material characterization

X-ray diffraction (XRD, Rigaku MiniFlex 600) patterns were obtained with a Cu Kα radiation source (λ = 1.5406 Å) at 40 kV. The morphology of the samples was studied by field-emission scanning electron microscopy (FESEM, FEI Quanta 200) with an operation voltage of 20 kV, and transmission electron microscopy (TEM, Tecnai G2 TF30 S-Twin) with an accelerating voltage of 300 kV. The X-ray photoelectron spectroscopy (XPS) measurements were carried out by a Thermo fisher Scientific K-Alpha$^+$ (Al Kα, 1486.6 eV) XPS spectrometer.

1.3 Electrochemical measurements

Electrochemical measurements were conducted on an Autolab electrochemical workstation using a standard three-electrode system. All experiments were carried out at room temperature in an O_2- or N_2-saturated 0.1 M KOH solution using a rotating disk electrode (RDE, 5 mm in diameter, 0.196 cm^2) as the working electrode. The Ag/AgCl (saturated KCl solution) and Pt plate were used as the reference electrode and counter electrode, respectively. All potentials in this study were converted into reversible hydrogen electrodes (RHEs) according to the following equation: (E_{RHE} = $E_{Ag/AgCl}$ + 0.059 × pH + 0.197). To prepare the working electrode, the ink was made by mixing 5 mg of the electrocatalyst with 50 µL Nafion solution (5 wt%) in 400 µL of DI water and 600 µL of ethanol, followed by 4h of sonication. Then, 20 µL of this electrocatalyst ink was pipetted onto the RDE and dried naturally (0.48 mg·cm^{-2}). The Tafel slopes were obtained by replotting the polarization curves as potential versus log|J| to assess the ORR and OER kinetics of the electrocatalysts.

(1) $$\frac{1}{J}=\frac{1}{J_L}+\frac{1}{J_K}=\frac{1}{B\omega^{1/2}}+\frac{1}{J_K}$$

(2) $$B=0.62\mathrm{n}FC_0(D_0)^{2/3\nu-1/6}$$

(3) $$J_K=nFkC_0$$

where J is the measured current density and the electrode-rotating rate (rad·s^{-1}). JL and JK are the diffusion- and kinetic-limiting current densities, n is the transferred electron number, F is the Faraday constant (F= 96485C mol^{-1}), C_0 is the O_2 concentration in the electrolyte (C_0= 1.2×10^{-6} mol cm^{-3}), D_0 is the diffusion coefficient of O_2 (D_0= 1.9×10^{-5} cm^2 s^{-1}), and v is the kinetic viscosity (v= 0.01 cm^2 s^{-1}). The constant 0.62 is adopted when the rotation speed is expressed in rad s^{-1}.

1.4 Zn–air battery

All Zn–air batteries were evaluated at room temperature. The electrocatalyst ink consisted of 5.0 mg electrocatalyst dispersed in 2.4 mL ethanol, 1.6 mL DI water, and 50 µL Nafion solution (5 wt%). The air electrode was prepared by uniformly coating the as-prepared electrocatalyst ink onto carbon paper (0.5 mg cm^{-2}) then drying naturally at room temperature. A Zn plate and electrocatalyst loaded onto carbon paper were used as the anode and cathode, respectively. The two electrodes were assembled into a homemade Zn–air battery, and 6 M KOH aqueous solution containing 0.2 M $Zn(CH_3COO)_2\cdot2H_2O$ was used as the electrolyte. All Zn–air battery tests were performed on a CHI660E electrochemical workstation with a LAND-CT2001A battery test system.

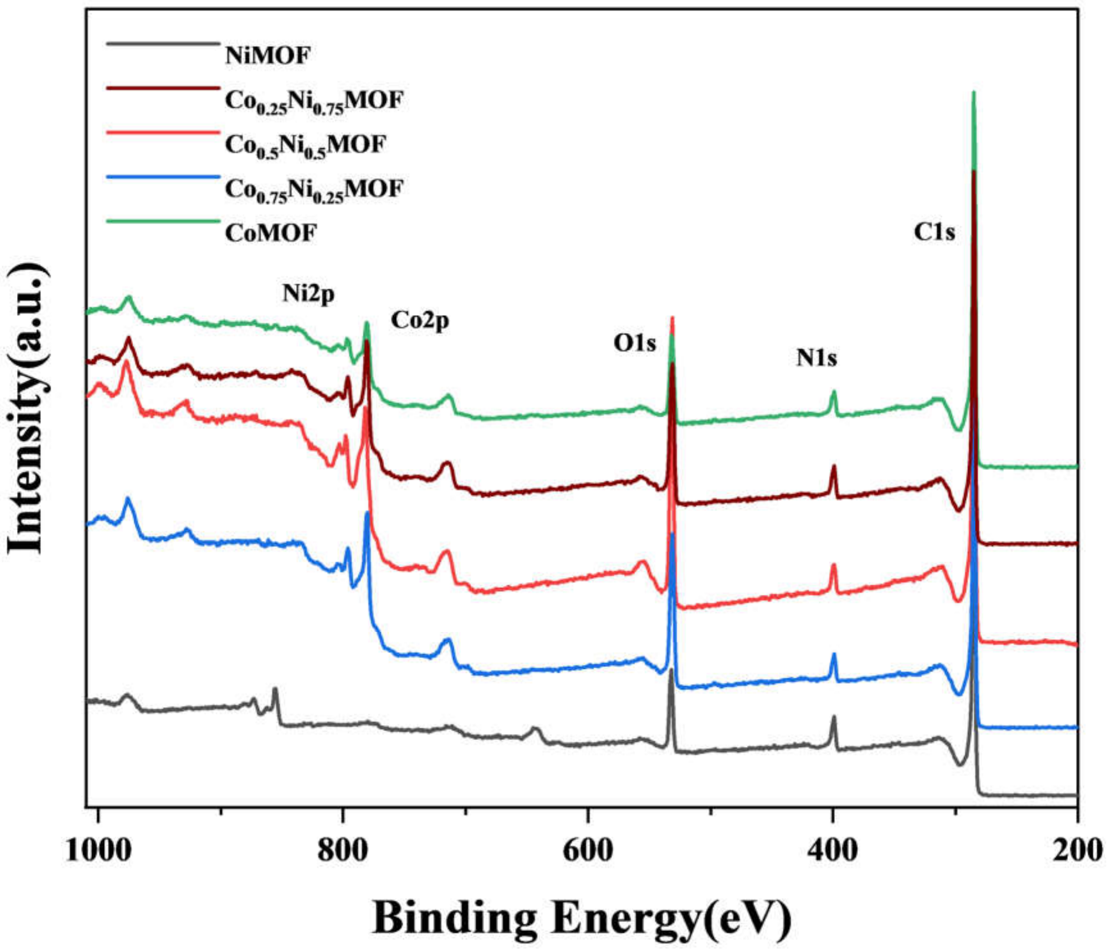

Figure S1. XPS spectra for Co_xNi_yMOF.

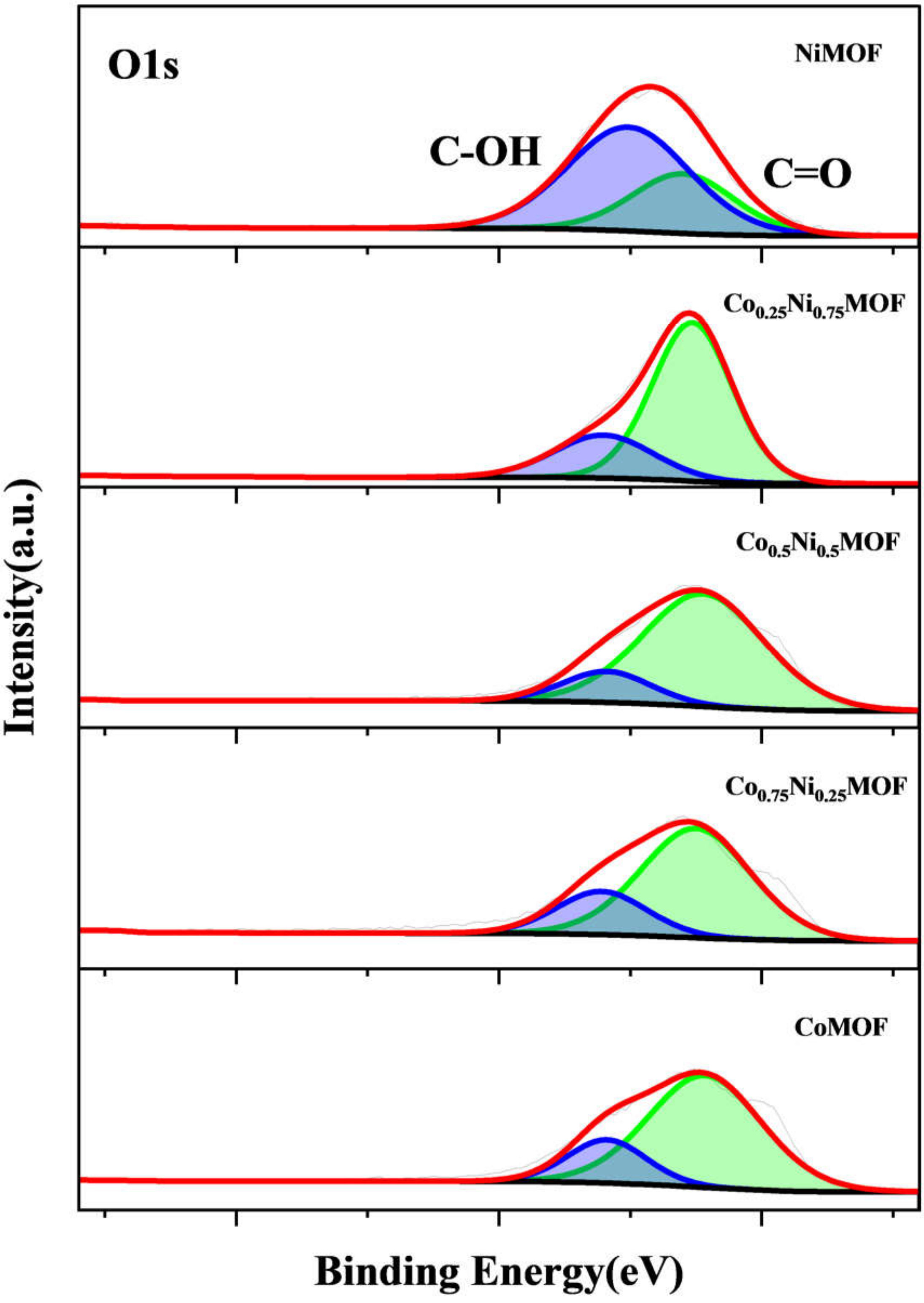

Figure S2. High-resolution XPS O 1s spectra of Co_xNi_yMOF.

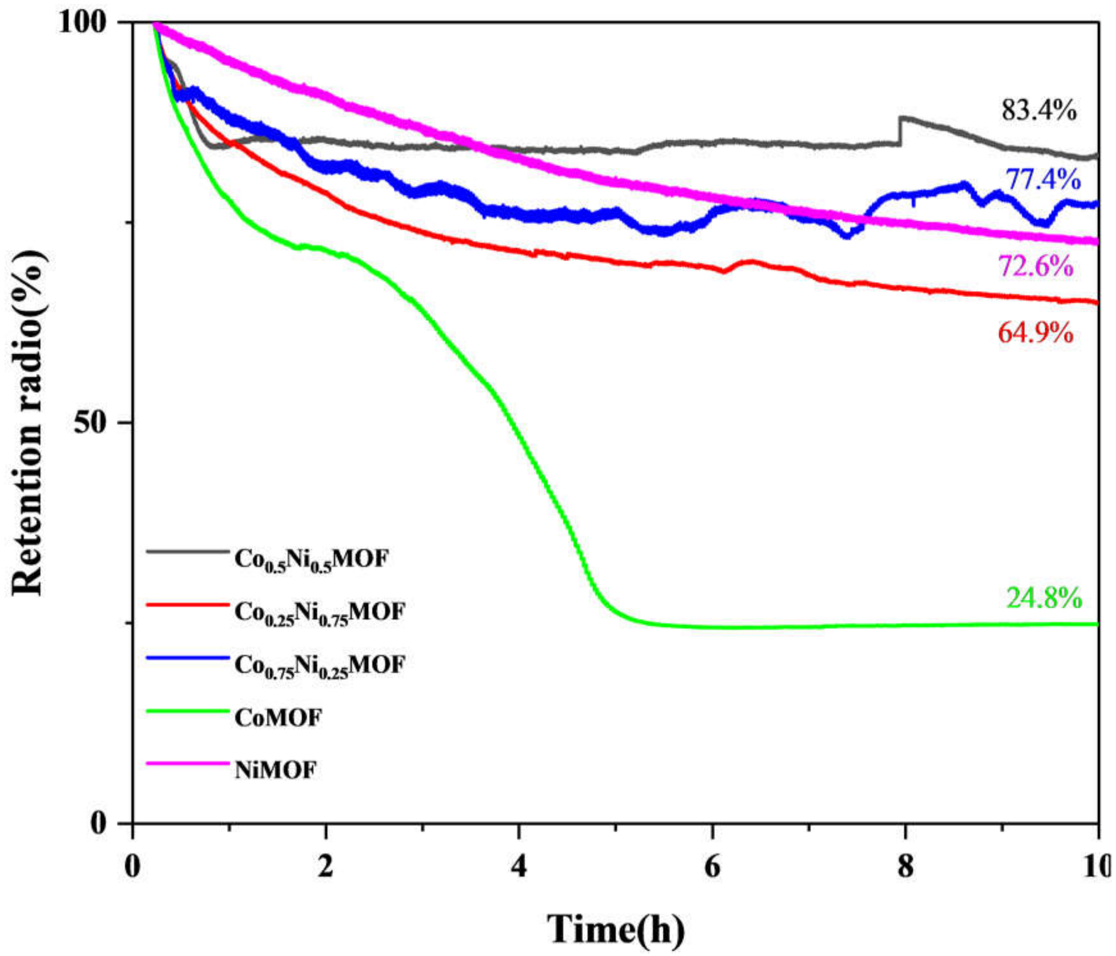

Figure S3. Current－time (i-t) curves of Co_xNi_yMOF.

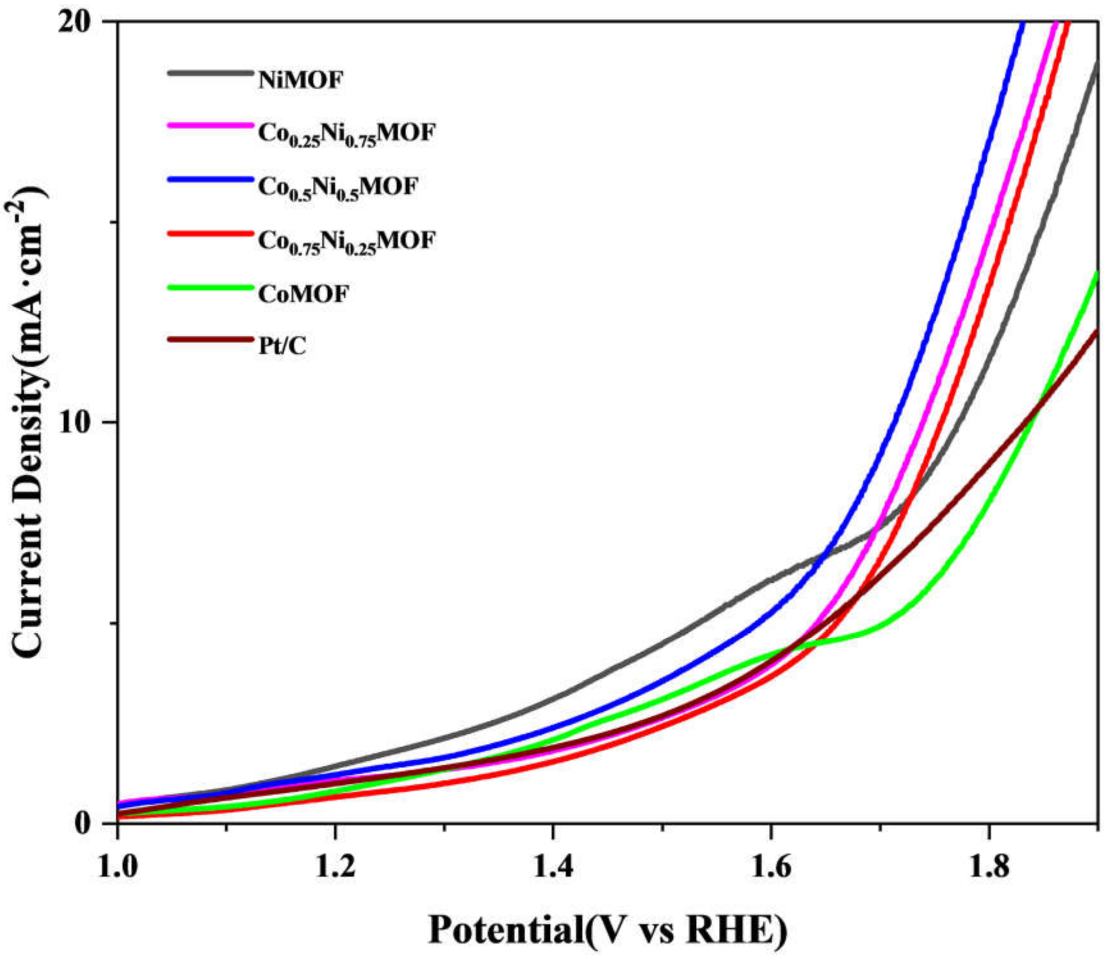

Figure S4. Polarization curves of Co_xNi_yMOF and Pt/C recorded at a scan rate of 10 mV s^{-1} in 0.1 M KOH solution..

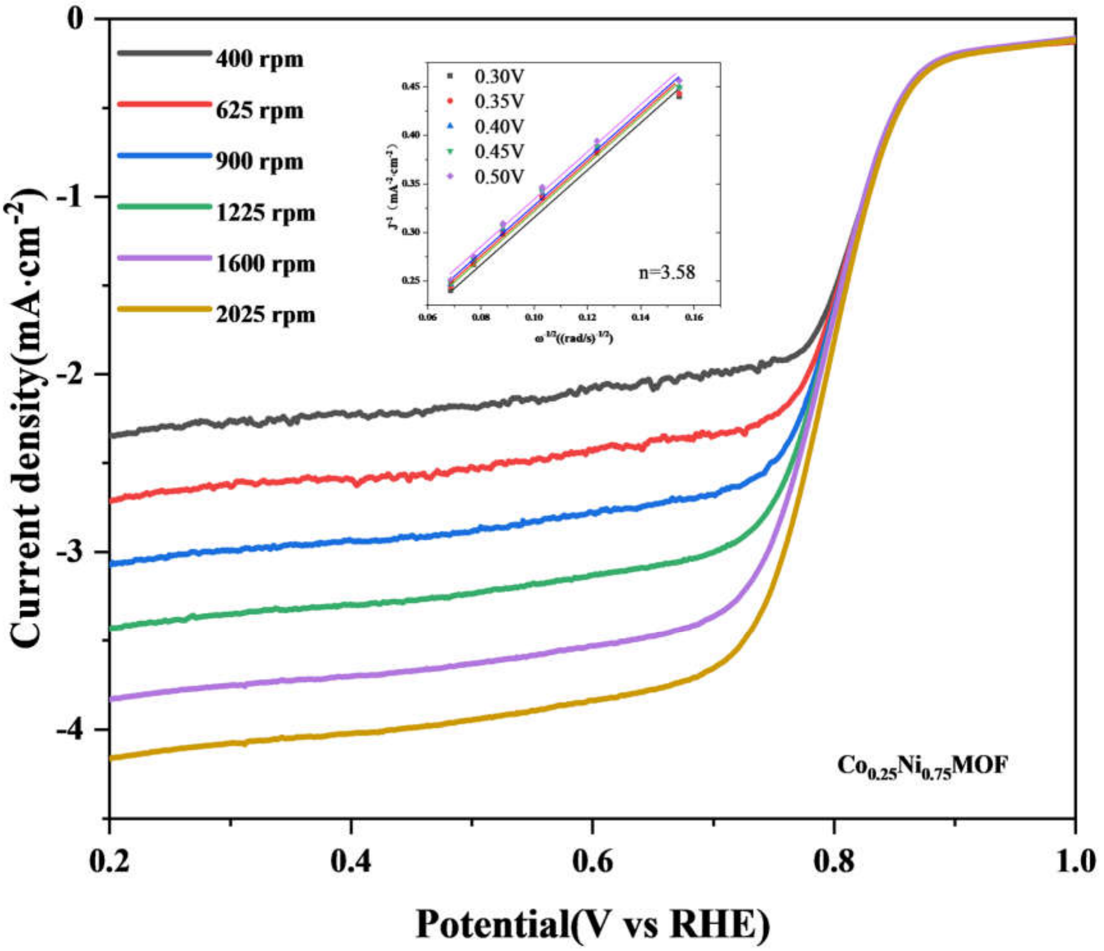

Figure S5. Koutecky－Levich plots of the $Co_{0.25}Ni_{0.75}MOF$ catalysts between 0.30 and 0.50 V.

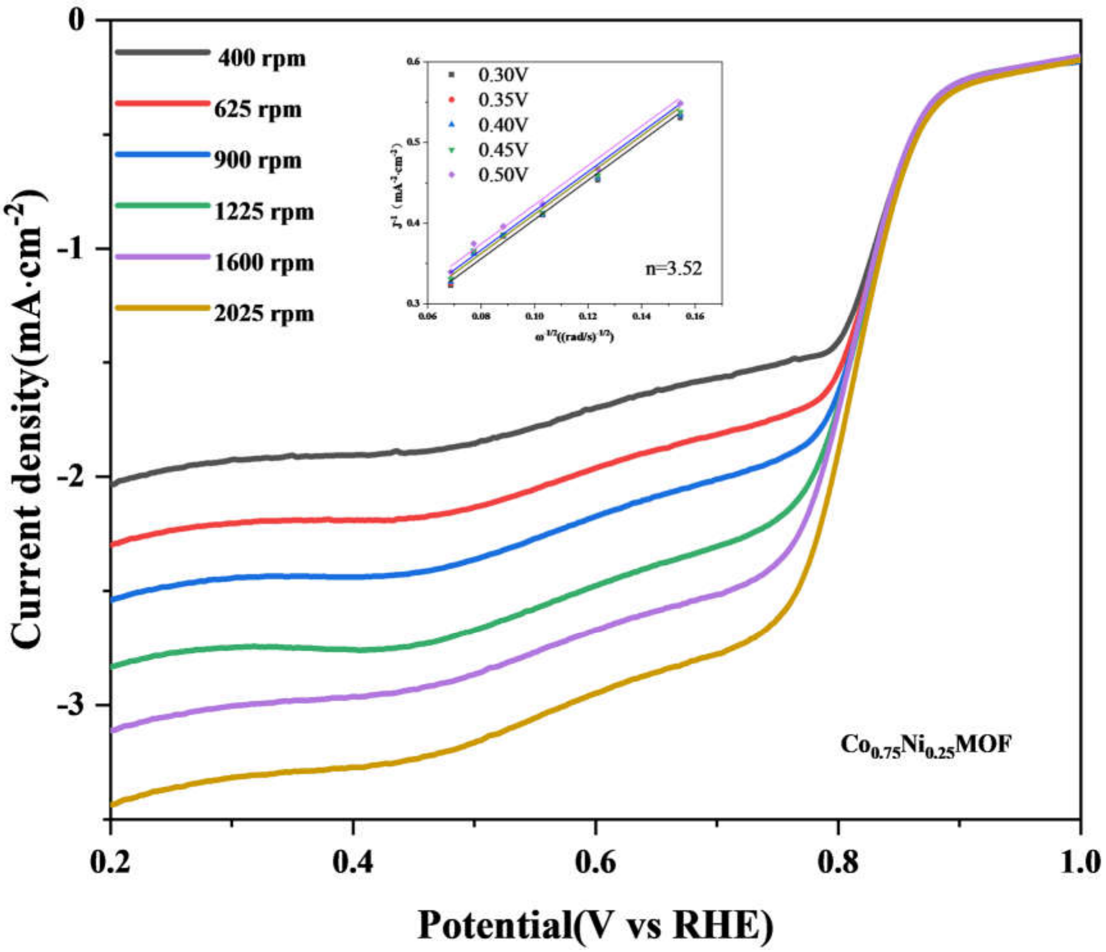

Figure S6. Koutecky–Levich plots of the $Co_{0.75}Ni_{0.25}$MOF catalysts between 0.30 and 0.50 V.

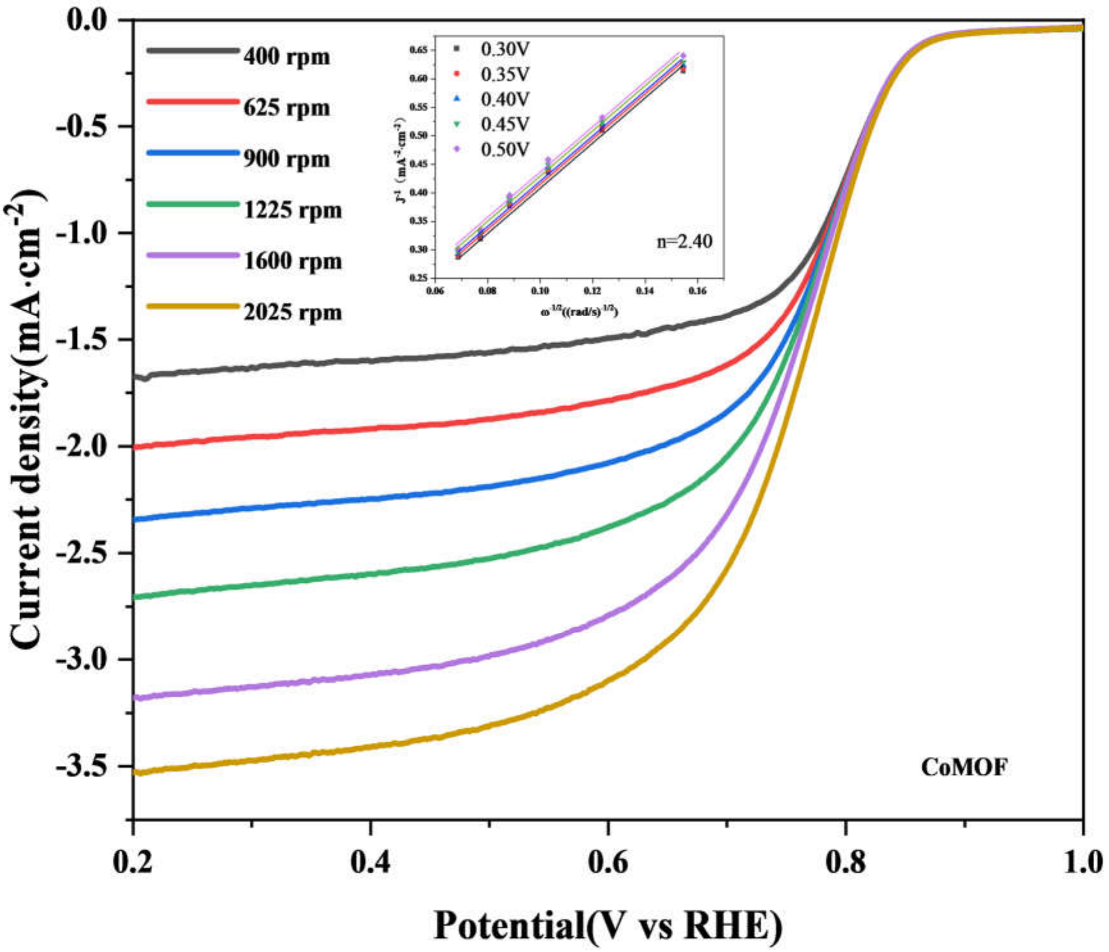

Figure S7. Koutecky‐Levich plots of the CoMOF catalysts between 0.30 and 0.50 V.

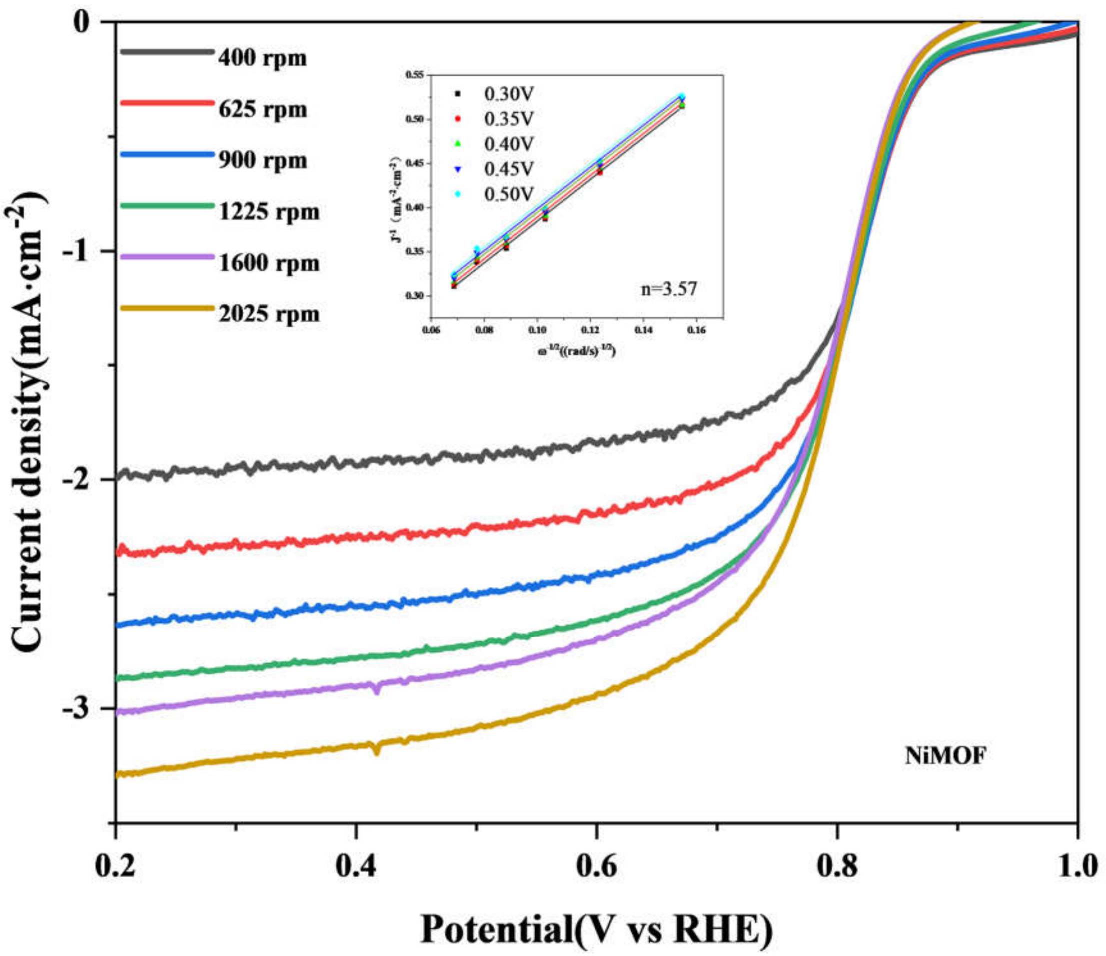

Figure S8. Koutecky − Levich plots of the NiMOF catalysts between 0.30 and 0.50 V.

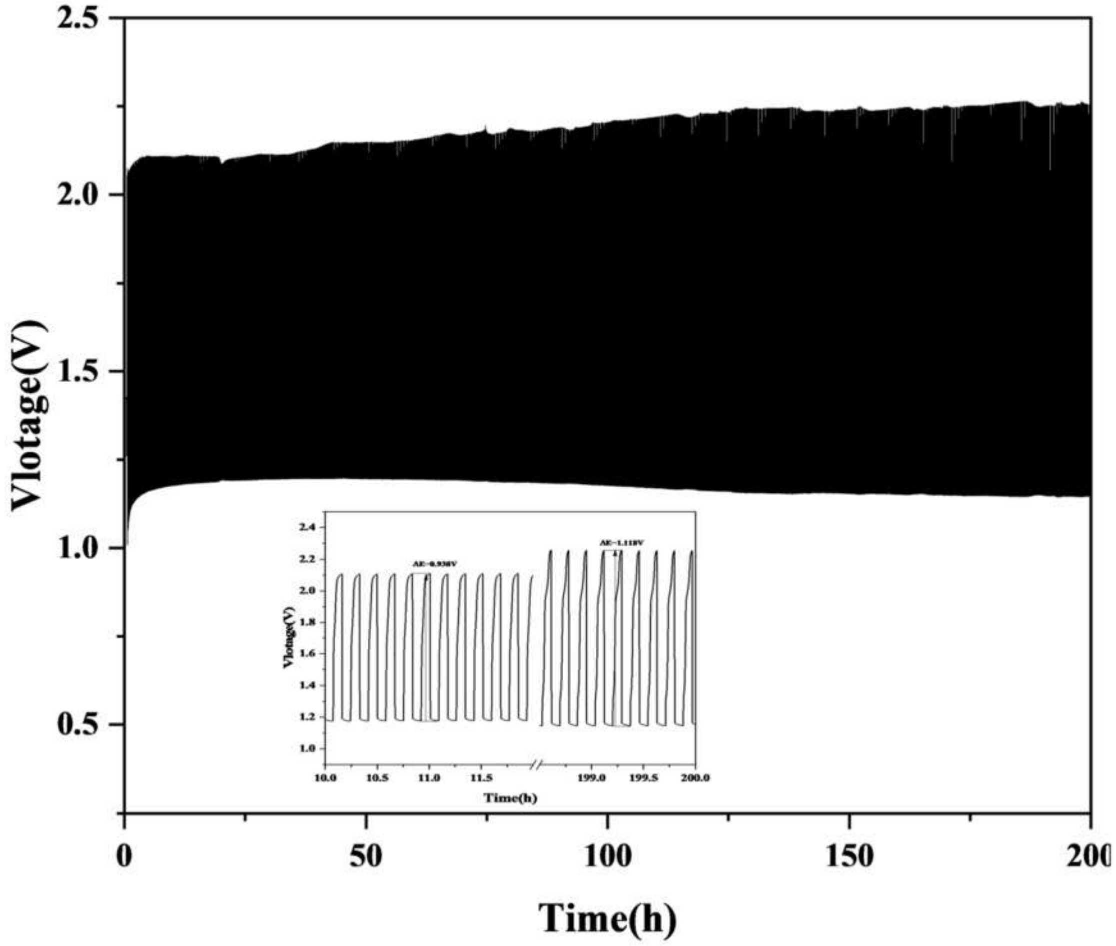

Figure S9. Charge–discharge curves of the CoMOF Zn－air batteries at 5 mA cm^{-2}.

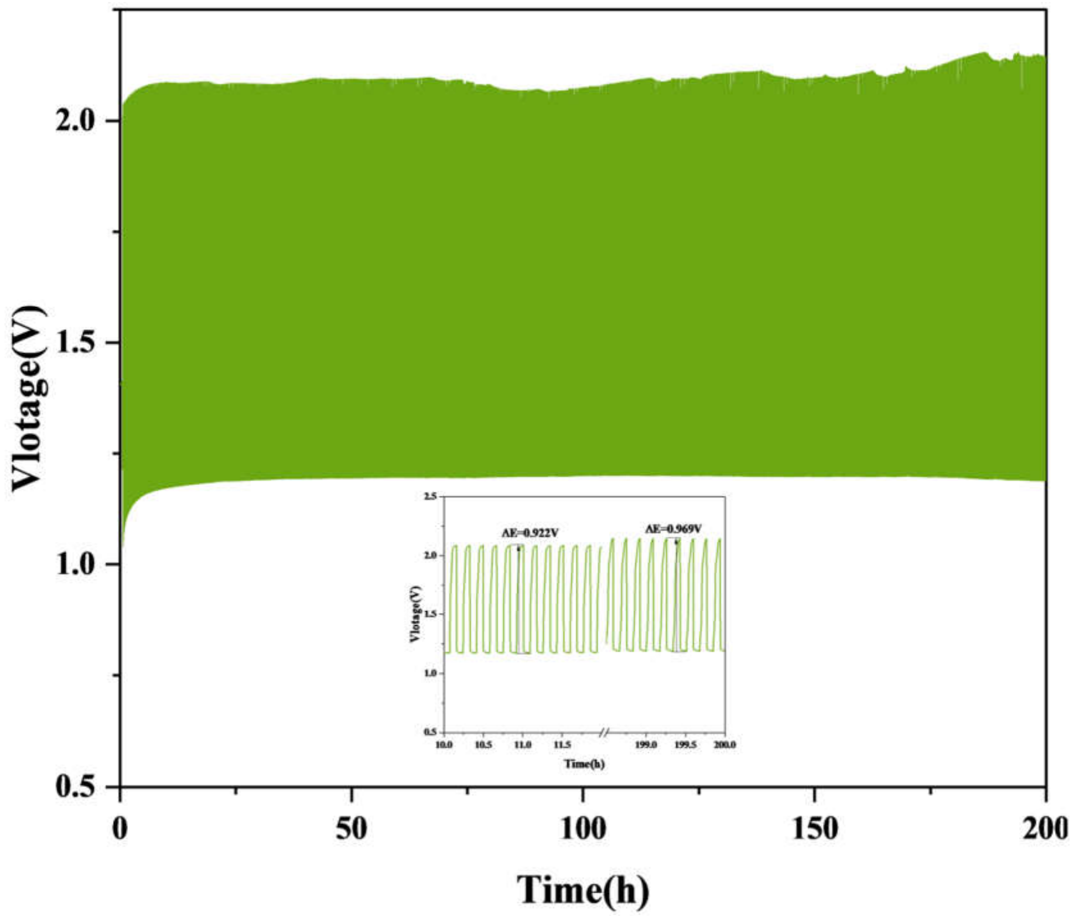

Figure S10. Charge–discharge curves of the $Co_{0.75}Ni_{0.25}$MOF Zn – air batteries at 5 mA cm^{-2}.

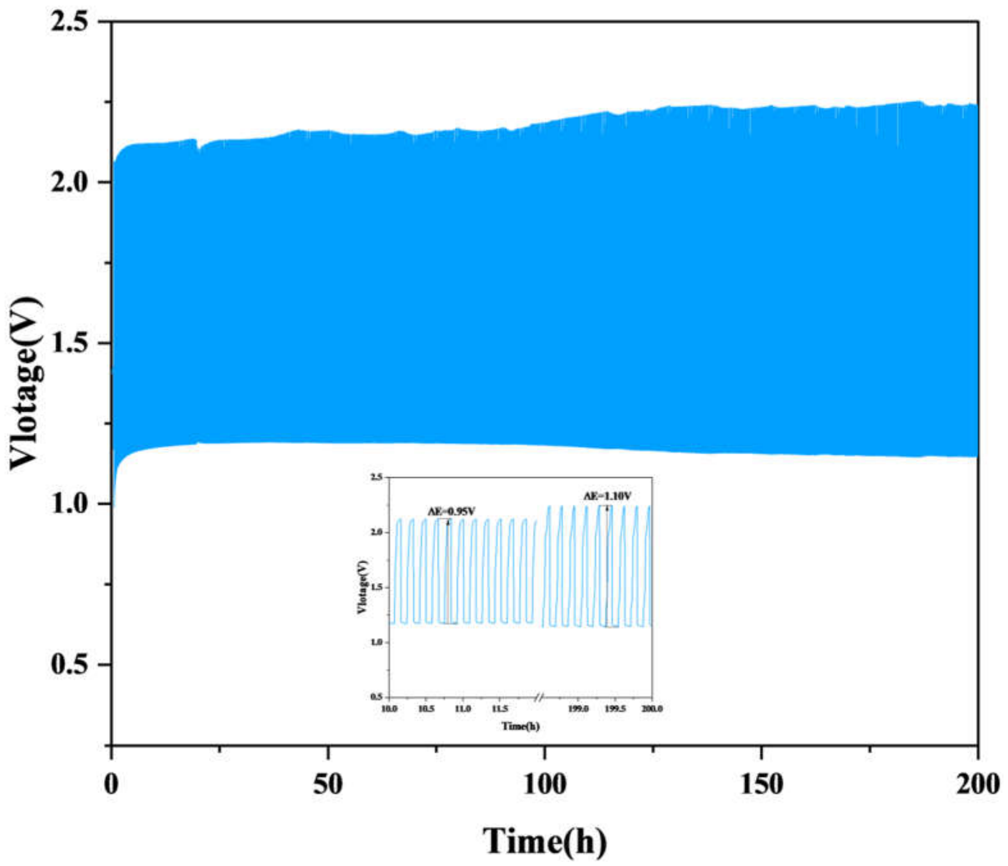

Figure S11. Charge–discharge curves of the $Co_{0.25}Ni_{0.75}MOF$ Zn－air batteries at 5 mA cm^{-2}.

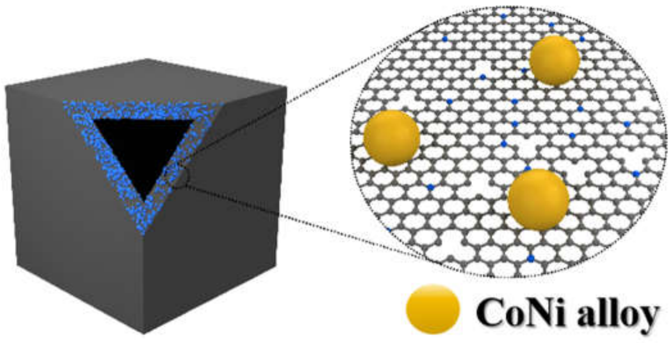

Figure S12. Schematic structure of $Co_{0.5}Ni_{0.5}MOF$ catalysts.

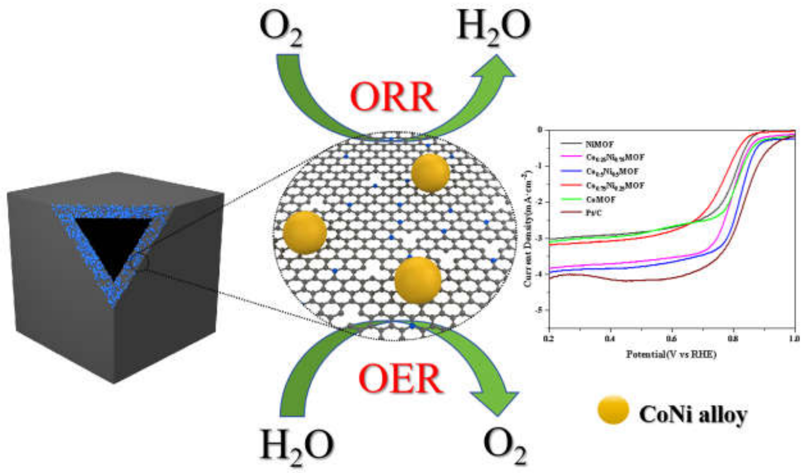

Figure S13. Schematic diagram of material structure and mechanism.

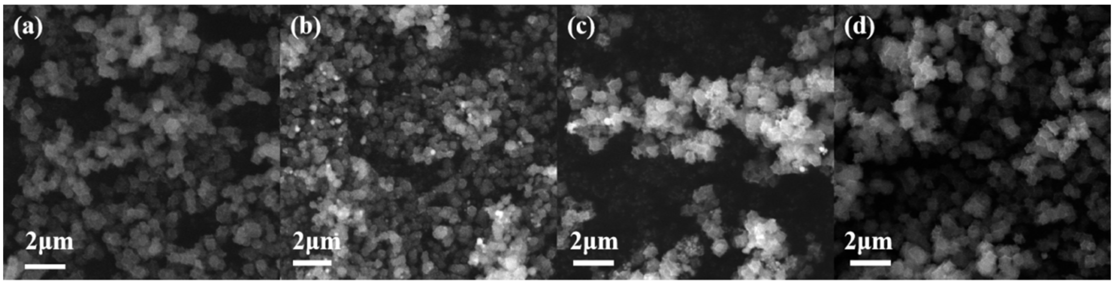

Figure S14. SEM images of (a) CoMOF, (b) $Co_{0.75}Ni_{0.25}$MOF, (c) $Co_{0.25}Ni_{0.75}$MOF, and (d) Ni MOF.

Table S1. Summary of ORR and OER performance of Co_xNi_yMOF electrocatalysts.

Electrocatalyst	ORR E_{onest} (V)	ORR $E_{1/2}$ (V)	ORR J ($mA\cdot cm^{-2}$)	OER η @ 10 $mA\cdot cm^{-2}$ (mV)
$Co_{0.5}Ni_{0.5}$MOF	0.90	0.82	4.0	470
$Co_{0.75}Ni_{0.25}$MOF	0.85	0.75	3.2	520
$Co_{0.25}Ni_{i0.75}$MOF	0.86	0.80	3.8	500
CoMOF	0.88	0.80	3.1	600
NiMOF	0.85	0.79	3.0	540

Table S2. The specific surface area of the prepared samples.

	specific surface area($m^2\cdot g^{-1}$)
CoMOF	196.5
$Co_{0.75}Ni_{0.25}$MOF	246.0
$Co_{0.5}Ni_{0.5}$MOF	278.5
$Co_{0.25}Ni_{0.75}$MOF	262.2

NiMOF	171.1

Table S3. The prepared samples analyzed according to the battery performance of zinc–ai r batteries.

	Power density (mW·cm^{-2})	Specific discharge capacity (mA h g_{Zn}^{-1})
CoMOF	127.9	581
$Co_{0.75}Ni_{0.25}MOF$	134.2	648
$Co_{0.5}Ni_{0.5}MOF$	138.6	740
$Co_{0.25}Ni_{0.75}MOF$	134.5	723
NiMOF	133.8	549

Table S4. Parameters for the degassing process of nitrogen adsorption and desorption tes ts.

Degas Conditions			
Evacuation rate	5.0 mmHg/s	Temperature ramp rate	10 ℃/min
Unrestricted evac. from	5.0 mmHg	Target temperature	30 ℃
Vacuum level	0.001 mmHg	Hold pressure	100 mmHg
Evacuation time	10 min		

Table S5. Setup parameters for the analytical process of nitrogen adsorption and desorpti on testing.

Analysis Conditions			
Adsorptive	Nitrogen @ 77.35 K		
Preparation Options			
Fast evacuation		Leak test	
Evacuation rate	2.0 mmHg/s	Leak test duration	120 s
Unrestricted evac. from	5.0 mmHg		
Vacuum setpoint	10 μmHg		
Evacuation time	0,2 hours		
Po and Temperature Options			
Measure Po in the Po tube for each isotherm point. Enter the analysis bath temperature			

below.	
Equilibration interval	10 s

Table S6. C, N, O, Co, and Ni content of each catalyst surface measured by XPS.

	C(at%)	N(at%)	O(at%)	Co(at%)	Ni(at%)
CoMOF	76.00	5.41	14.13	4.45	0
$Co_{0.75}Ni_{0.25}MOF$	71.85	4.08	19.38	4.55	0.14
$Co_{0.5}Ni_{0.5}MOF$	74.19	4.57	16.86	4.22	0.16
$Co_{0.25}Ni_{0.75}MOF$	81.55	5.20	10.22	2.91	0.12
NiMOF	81.31	6.91	9.96	0	1.81

Table S7. Relative content of C=C, C-N, and C-O bonds to elemental C in each catalyst s urface.

	C=C(at%)	C-N(at%)	C-O(at%)
CoMOF	62.9	17.6	19.5
$Co_{0.75}Ni_{0.25}MOF$	62.5	22.8	14.7
$Co_{0.5}Ni_{0.5}MOF$	62.5	23.6	13.9
$Co_{0.25}Ni_{0.75}MOF$	63.3	20.2	16.5
NiMOF	63.1	17.4	19.5

Table S8. C-OH and C=O bonds accounting for the relative content of elemental O in ea ch catalyst surface.

	C-OH(at%)	C=O(at%)
CoMOF	23.5	76.5
$Co_{0.75}Ni_{0.25}MOF$	26.2	73.8
$Co_{0.5}Ni_{0.5}MOF$	18.3	81.7
$Co_{0.25}Ni_{0.75}MOF$	26.5	73.5
NiMOF	68.4	31.6

Table S9. The content of different types of N on the surface of the prepared catalysts o btained by XPS tests.

	Pyridine N (at%)	Pyrrole N (at%)	graphitic N (at%)	Oxide N (at%)
CoMOF	33	28	27	12
$Co_{0.75}Ni_{0.25}MOF$	25	39	27	9
$Co_{0.5}Ni_{0.5}MOF$	35	30	25	10
$Co_{0.25}Ni_{0.75}MOF$	44	15	28	13
NiMOF	43	22	24	11

Table S10. The molar ratios of cobalt nitrate and nickel nitrate used in the synthesis pro cess of different samples.

	Cobalt nitrate（g）	Nickel nitrate（g）
CoMOF	1.63	0
$Co_{0.75}Ni_{0.25}MOF$	1.222	0.407
$Co_{0.5}Ni_{0.5}MOF$	0.815	0.814
$Co_{0.25}Ni_{0.75}MOF$	0.407	1.221
NiMOF	0	1.628

Table S11. The molar ratios of cobalt nitrate and nickel nitrate used in the synthesis pro cess of different samples.

	Cobalt nitrate（%）	Nickel nitrate（%）
CoMOF	100	0
$Co_{0.75}Ni_{0.25}MOF$	75	25
$Co_{0.5}Ni_{0.5}MOF$	50	50
$Co_{0.25}Ni_{0.75}MOF$	25	75
NiMOF	0	100

Table S12. The specific surface area of pore volumes.

	t-Plot micropore volume($cm^3 \cdot g^{-1}$)
CoMOF	0.0196
$Co_{0.75}Ni_{0.25}MOF$	0.0309
$Co_{0.5}Ni_{0.5}MOF$	0.0307
$Co_{0.25}Ni_{0.75}MOF$	0.0100
NiMOF	0.0229
